# Effects of taxane-anthracycline and taxane only treatment on cardiac function in breast cancer—a retrospective cohort study

**DOI:** 10.1186/s40959-025-00335-4

**Published:** 2025-04-12

**Authors:** Árpád Kézdi, Emese Szelke, Magdolna Dank, Dorottya Mühl, Gyöngyvér Szentmártoni, Gergely Szabó, Dominic Joseph Fogarasi, István Takács, Viktor J Horváth, Ádám G Tabák

**Affiliations:** 1 Department of Internal Medicine and Oncology, Semmelweis University, Budapest, Hungary; 2 School of PhD Studies, Semmelweis University Faculty of Medicine, Budapest, Hungary; 3 Institute of Preventive Medicine and Public Health, Semmelweis University Faculty of Medicine, Budapest, Hungary; 4 Department of Epidemiology and Public Health, University College London, London, UK

**Keywords:** Breast cancer, Cardiotoxicity, Anthracycline, Taxane, Echocardiography

## Abstract

**Introduction:**

Cardiotoxic, anthracycline-based therapies have high value in selected patients with breast cancer. We aimed to describe the effect of anthracycline plus taxane and single taxane chemotherapies on echocardiographic parameters in women with breast cancer.

**Methods:**

We retrospectively analysed data of 68 women (> 18 years old) treated for breast cancer in 2018–2021 in the Cardiology Outpatient Clinic of Semmelweis University, Department of Internal Medicine and Oncology. Cardiovascular medical history was collected at baseline and transthoracic echocardiography was completed at each visit. Also, we reviewed electronic medical records for other relevant medical information. Measured echocardiography parameters were assigned to five periods (0–14 days, then every half year and beyond day 545) based on the time since the first treatment. Trajectories of ejection fraction and diastolic function associated markers over the follow-up periods were analysed by linear mixed models.

**Results:**

Mean age of the anthracycline plus taxane group was 52.7 ± 14.1 years, of the single taxane group 55.2 ± 13.1 years. The mean anthracycline dose was equivalent to 240 mg/m^2^ of doxorubicin. Overall pre-existing cardiovascular burden was low. Statistically significant changes were found only in the anthracycline plus taxane group: ejection fraction decreased mildly from 65.5 ± 3.1% at baseline to 62.1 ± 3.2% at 181–365 days (*p* = 0.007) while deceleration time decreased mildly from 227.9 ± 33.9 msec to 197.4 ± 29.4 msec at 15–180 days (*p* = 0.028). Both drops were only temporary and values neared baseline values over follow-up (p = NS vs. baseline). Other important determinants of ejection fraction were age and hypertension among the investigated risk factors.

**Conclusion:**

Our study confirms the overall safety on cardiac function of both single taxane and anthracycline plus taxane chemotherapy, as we found no changes in echocardiographic parameters associated with single taxane therapy, while anthracycline plus taxane chemotherapy was associated with a temporary and clinically insignificant reduction of ejection fraction and deceleration time over 1.5 years of follow-up. Our study is limited by its retrospective nature and the low number of participants.

**Supplementary Information:**

The online version contains supplementary material available at 10.1186/s40959-025-00335-4.

## Introduction

Breast cancer is one of the most diagnosed malignancies in the world and among females it is the leading cause of cancer-related mortality with approximately 685 000 deaths worldwide in 2020 [1]. With advanced, combined therapies the overall survival is increasing and nearly 90% of women at any stage survive the diagnosis by at least 5 years and 75% by 10 years [2].

Cardiovascular disease (CVD) associated death is the leading non-cancer related cause of mortality among women worldwide [3]. Risk factors for CVD have been associated with the development of breast cancer [4, 5]; likewise, patients with breast cancer commonly present with multiple CV risk factors [6]. As the bimodal age distribution of breast cancer suggests, patients with fewer CV risk factors are also affected by breast cancer [7, 8], but there is a clear synergistic effect between the development of CVD and breast cancer. Furthermore, some of the conventional chemotherapies applied first line in patients with breast cancer are known to have cardiotoxic side effects [2]. This may explain why CVD (and not cancer itself) is the primary cause of death in patients aged ≥ 75 years with stage I or localized breast cancer [9, 10]. These data, together with the increasing survival and age of surviving patients highlight the need for cardiac monitoring during and after oncological treatment.

Identifying the stage and molecular subtype of breast cancer is crucial for choosing proper treatment. In selected cases, no chemotherapy is required. However, if chemotherapy is recommended, anthracyclines (mainly doxorubicin and epirubicin) and taxanes (paclitaxel, docetaxel) are frequently recommended and have high value in selected clinical situations, although the therapy of many patients with breast cancer does not include anthracyclines. Taxanes are usually considered to be safe from a cardiological standpoint, but anthracyclines have pronounced cardiac side effects. Cardiac events and death increase approximately fivefold after anthracycline-based versus non-anthracycline regimens [11]. Anthracycline‐related cardiac events typically occur within the first year [12], although long-term cardiac damage and remodelling have also been described [13]. Therefore, early detection of cardiac abnormalities during treatment is crucial.

In 2022, the European Society of Cardiology (ESC) published its first guideline on cardio-oncology, with evidence levels and grades of recommendation [14]. The guideline covers the diagnosis, management and prevention of cardiovascular toxicity associated with cancer therapy for fifteen types of chemotherapy treatments. While the ESC guideline lists only anti-Her-2 and anthracycline-based treatments as harmful from a cardiological standpoint, other guidelines also recommend follow-up for the cardiac effects of taxane-based regimens [2].

In the Cardiology Outpatient Clinic of the Department of Internal Medicine and Oncology, Semmelweis University, Budapest, Hungary, the most frequently used chemotherapeutic agents in breast cancer were anthracyclines and anti-HER-2 (drugs with pronounced cardiac side effects), as well as taxanes and aromatase inhibitors (drugs with less pronounced cardiac side effects). The aim of our retrospective analysis was to describe the cardiac effects of anthracycline plus taxane treatment as well as single taxane therapy. In designing the analysis, we followed the ESC recommendations in defining both predictors and outcomes.

## Materials and methods

### Setting

This retrospective study was conducted on women > 18 years old diagnosed with breast cancer in the Department of Internal Medicine and Oncology, Semmelweis University, Budapest, Hungary, between April 2018 and April 2023. The study was conducted in accordance with the Declaration of Helsinki. Oral and written informed consent was obtained from all participants prior to inclusion in the study. Ethical approval was obtained from the Ethical Committee of Semmelweis University (RKEB/34/2021).

Patients with any stage of breast cancer requiring chemotherapy are regularly examined by a cardiologist. These examinations are carried out either at the Cardiology Outpatient Clinic or at an external hospital according to patient’s choice. Patients followed up in the clinic were asked to fill in a questionnaire on medical history and medications. Their weight and height were measured, and they received a transthoracic echocardiogram (TTE) examination at each visit.

In our study we reviewed the electronic medical records of patients examined at our clinic between April 2018 and December 2021. We selected patients treated with either a single taxane or an anthracycline plus taxane regimen for breast malignancy. For all eligible patients, we collected additional data from electronic records and performed a statistical analysis. Results of those tests performed at external hospitals were not collected.

### Selection of participants

Eligibility was determined based on data collected from the electronic medical records of the University. Inclusion criteria were female sex, age > 18 years, diagnosis of a breast malignancy (diagnosis including stage and molecular status according to the current protocol of the Hungarian College of Radiotherapy and Oncology) [15], and receipt of treatment with a single taxane (group 1) or an anthracycline plus taxane (group 2) chemotherapy. Anthracycline dose was based on the treating oncologists’ discretion in accordance with the most recent local guidelines and protocols [15]. Most patients were treated with 240 mg/m^2^ cumulative dose of doxorubicin or 300 mg/m^2^ cumulative dose of epirubicin (equivalent to 240 mg/m2 doxorubicin) and 80 mg/m^2^ paclitaxel. It should be noted that according to national guidelines anthracycline treatment is contraindicated for patients previously treated with cardiotoxic chemotherapies and thus no patients in the anthracycline plus taxane group received prior chemotherapy, while approximately a quarter of those on single taxane therapy received any prior chemotherapies. (Table 1) Exclusion criteria were the lack of a baseline visit (visit 0– within 14 days of chemotherapy treatment) or at least one visit after the baseline visit, and other prior chemotherapy within 365 days. Baseline visit was defined as a visit that took place before or within 14 days of the start of the first chemotherapy treatment.

**Table 1:** Baseline characteristics of participants by exclusion and by type of treatment

Variable	By exclusion			By type of treatment		
	Excluded	Included	*p*	Group 1	Group 2	*p*
n	300	68	-	45	23	-
Age (years)	57.1 ± 13.3	54.3 ± 13.4	0.116	55.2 ± 13.1	52.7 ± 14.1	0.475
BMI (kg/m2)	27 ± 5.6	25.7 ± 4.8	0.069	25.7 ± 4.8	25.7 ± 5	0.983
Hypertension	118/300 (39.3%)	24/68 (35.3%)	0.583	17/45 (37.8%)	7/23 (30.4%)	0.602
Diabetes mellitus	41/300 (13.7%)	6/68 (8.8%)	0.322	3/45 (6.7%)	3/23 (13%)	0.399
Hyperlipidaemia	36/300 (12%)	8/68 (11.8%)	1	3/45 (6.7%)	5/23 (21.7%)	0.109
Current smoking	44/300 (14.7%)	13/68 (19.1%)	0.357	8/45 (17.8%)	5/23 (21.7%)	0.75
MACE	14/300 (4.7%)	2/68 (2.9%)	0.746	0/45 (0%)	2/23 (4.3%)	0.111
**Previous chemotherapy**	**-**	**-**	**-**	**12/45 (26.7%)**	**0/23 (0%)**	**0.006**
EF (%)	-	-	-	64.3 ± 4.2	65 ± 4.7	0.527
DT (msec)	-	-	-	212.5 ± 1.3	229.1 ± 1.2	0.225
E/e’	-	-	-	6.5 ± 1.3	6.4 ± 1.3	0.834

Data are mean ± SD or n/total (%). p values based on independent samples t-tests or chi-square tests. *p* < 0.05 was considered as statistically significant. Group 1 included patients who received taxane-based chemotherapy treatment without anthracycline. Group 2 included patients who received anthracycline followed by taxane-based treatment. BMI, body mass index; MACE, major adverse cardiac events (prior acute myocardial infarction (ICD-10: I21x, I22x) or unstable angina (ICD-10: I200), ischaemic or haemorrhagic stroke (ICD-10: I60x-I64x); peripheral arterial disease (ICD-10: I738, I739)); EF, ejection fraction; DT, deceleration time; E/ e’, ratio of early diastolic flow peak velocity of the mitral valve (E) and early diastolic peak velocity of mitral valve annulus (e’). Hypertension, hyperlipidaemia and diabetes mellitus were diagnosed based on medication use (at least one drug for their treatment) and the diagnosis was coded in the medical record at least three times by a proper ICD-10 code (hypertension: I10xx, hyperlipidaemia: E78xx; diabetes mellitus: E10xx, E11xx or E14xx)). Previous chemotherapy was defined as anthracycline or anti-HER2 treatment beyond 365 days. Of the 12 patients, 10 received anthracycline chemotherapy and 6 received Anti-HER2 therapy

**Fig. 1:**
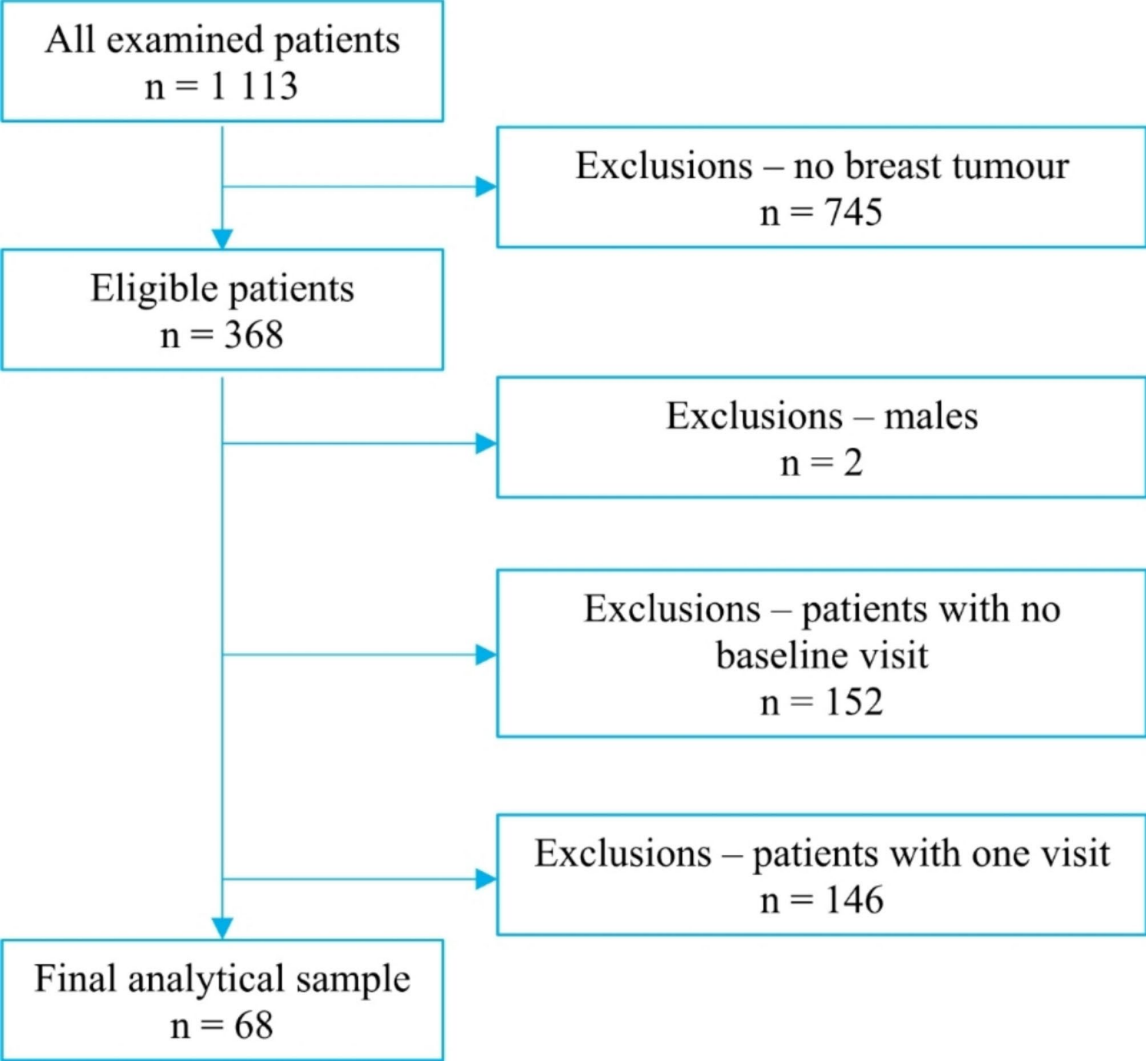
Flow chart of patient selection

Between April 2018 and December 2021, a total of 1 113 oncology patients were treated in the Department of Internal Medicine and Oncology of Semmelweis University and underwent at least one cardiology examination at the Cardiology Outpatient Clinic of the Department. Of these 1 113 patients, 368 patients (33.06%) were diagnosed with breast malignancy. Of these 368 patients we excluded 2 men (0.54% of patients with breast malignancy). Further 152 patients (41.30%) were excluded due to missing baseline visit. Finally, we excluded 146 (39.67%) patients because they had no follow-up data. Thus, our final analytical sample represents data of 68 patients (18.5%) with 332 visits (28.1%). (Fig. 1**)**

### Baseline assessment and predictors

Following testing for eligibility, further data were collected. Patients examined in our clinic completed a questionnaire related to their cardiovascular medical history. Information on smoking history was recorded from questionnaires completed by patients. Body weight, height and blood pressure were measured with calibrated instruments at each examination. Body weight was measured in light clothing without shoes on a calibrated digital scale (ADE M320000-01 electronic weight scale, ADE, Germany), height was measured without shoes and was rounded to the nearest centimetre [16]. BMI was calculated as weight (kg)/height^2^ (m). Resting sitting blood pressure was measured twice using a calibrated digital meter (OMRON M3, Omron, Japan) after a 5-minute rest. The average of the two measurements was used.

In addition to the above data, we also used electronic records of the University to collect data on cardiovascular comorbidities, including hypertension, diabetes mellitus, hyperlipidaemia, and major adverse cardiovascular events (MACE, defined as prior acute myocardial infarction (ICD-10: I21x, I22x) or unstable angina (ICD-10: I200), ischaemic or haemorrhagic stroke (ICD-10: I60x-I64x); peripheral arterial disease (ICD-10: I738, I739)). Hypertension, hyperlipidaemia and diabetes mellitus were diagnosed based on medication use (at least one drug for their treatment) and the diagnosis was coded in the medical record at least three times by a proper ICD code (hypertension: I10xx, hyperlipidaemia: E78xx; diabetes mellitus: E10xx, E11xx or E14xx). Additional information was collected from electronic records on the dates and types of chemotherapy treatments and other medications, and the results of TTE examinations. Previous chemotherapy was defined as anthracycline or anti-HER2 treatment beyond 365 days.

These data were also collected through the patient questionnaires. After comparing questionnaires with medical records, any discrepancies were discussed with the patients and the data was rectified in the database.

Finally, we stratified the patients into two groups based on the treatment regimen. Group 1 included patients who received single taxane chemotherapy treatment without anthracycline. Group 2 included patients who received anthracycline followed by taxane-based treatment.

### TTE outcomes and time periods

A TTE was performed during each visit with the same Siemens Acuson X300 machine and P5-1 transducer (Siemens Hungary KFT, Budapest) [17, 18].

During each visit an expert echocardiographer (VH, ES, ÁK, GS) completed the TTE examination. Echocardiographic measurements were performed according to the current ESC recommendations [19]. Echocardiographers were unaware of the chemotherapy regimen used and thus could be considered blinded. Results were discussed with other specialists only in cases of inconsistencies (e.g., discrepancies between markers characterizing systolic or diastolic function). The following parameters were measured at least 3 times, and the average was calculated for analysis: left ventricle ejection fraction with Simpson’s method (biplane method of disks by using the apical four-chamber and two-chamber views; EF; %), deceleration time (from apical four chamber view, calculated as the time between the peak of E wave and the upper deceleration slope extrapolated to zero line; DT; msec), early diastolic flow peak velocity of the mitral valve (apical four chamber view; E; cm/sec) and early diastolic peak velocity of the mitral valve annulus (apical four chamber view; average of medial and lateral velocities; e’; cm/sec). To estimate diastolic performance of the heart, DT and the ratio of E and e’ (E/e’) were used.

Follow-up was divided into 5 discrete periods based on the time elapsed from the first treatment to be able to describe outcome trajectories. The periods were the following:

- Period 0: data measured within 14 days (at baseline visit),
- Period 1: data measured between 15 and 180 days,
- Period 2: data measured between 181 and 365 days,
- Period 3: data measured between 366 and 545 days,
- Period 4: data measured beyond 546 days.

### Statistical analysis

Data were visually inspected for normality as well as by normality tests. Because of the skewed distribution of the DT and E/e’ values, we used their natural logarithms.

Descriptive statistics were provided for all baseline characteristics. Normally distributed data (like age, BMI, EF) were presented as means ± SD, while non-normally distributed data were expressed as median (interquartile range). Categorical data were reported as n (%). For the comparison of included and excluded patients, as well as of single taxane group and anthracycline plus taxane group, independent sample t-tests for continuous variables and χ^2^ tests for categorical data were used.

Given that each patient provided multiple measurement points for analysis, outcomes (differences in TTE parameters between periods) were analysed by linear mixed models. Three hierarchical models were run for each TTE outcome. *Model 1* included the type of chemotherapy, the periods, and their interaction as fixed effects. *Model 2* additionally included all risk factors as fixed effects (age, BMI, hypertension, diabetes mellitus, hyperlipidaemia, current smoking, MACE, previous chemotherapy). Finally, for the third model we excluded in a stepwise manner all risk factors that were statistically not significant from the second model to reach the most parsimonious model (*Model 3*). For easier interpretation, we report results only for *Model 2* in the main manuscript, and results of *Model 1* and *Model 3* are presented in supplementary tables.

As most patients were on concurrent anti-HER2 treatment (40/45 patients in single taxane group and 18/23 patients in anthracycline plus taxane group) we also run a sensitivity analysis limited to participants that received anti-HER2 therapy in addition to their chemotherapy (single taxane or anthracycline plus taxane). Given the limited number of participants in this analysis, we only run *Model 1*.

A 2-sided *p* < 0.05 was considered statistically significant. All analyses were performed using SPSS version 28.0.

## Results

### Baseline characteristics

Included and excluded patients were similar in those baseline characteristics (age, BMI, presence of hypertension, diabetes mellitus, and hyperlipidaemia, current smoking, previous MACE) that were collected (all *p* > 0.05), suggesting no major selection bias in these parameters. (Table 1)

Baseline characteristics (including TTE parameters) of the two treatment groups were also similar except for more frequent presence of a previous chemotherapy in the single taxane group (group 1–26.7% vs. group 2–0%, *p* = 0.006). (Table 1) Of those 12 patients on single taxane therapy that received previous chemotherapy, 10 received anthracycline and 6 anti-HER2 therapy. Previous chemotherapy was given a median 2 791 days before baseline (range: 707–5 107 days).

In single taxane group, 45 patients had a total of 228 visits, in anthracycline plus taxane group, 23 patients had 104 visits. All patients had at least two visits, with the highest number of visits being 23 for single taxane and 17 for anthracycline plus taxane group. Stratified by the periods, all patients had a baseline visit (group1/group2: 45/23), 40/24 visits in period 1, 55/21 visits in period 2, 37/14 visits in period 3, and 51/22 visits in period 4.

### Transthoracic echocardiography

In the single taxane group no significant change compared to baseline was found in any of the TTE parameters based on *Model 2*, although one patient’s EF dropped from a baseline of 63–49% in period 4. On further follow-up the EF returned to normal even in this case. Exclusion or inclusion of this patient did not alter the results. (Table 2) *Model 1* and *Model 3* provided very similar results. (Supplementary Table 1)

**Table 2:** Estimated marginal means of EF, DT, E/e’ by treatment type based on *Model 2* over the follow-up periods

Days	Model 2		
	EMM (95% CI)	Diff	*p*
**Group 1 - EF (%)**			
0–14	65.6 (62.6;68.7)	0 (ref).	-
15–180	64.8 (61.7;67.9)	-0.8	0.431
181–365	64.4 (61.4;67.4)	-1.2	0.146
366–545	66.7 (63.5;69.8)	1.0	0.193
> 545	66.5 (63.4;69.7)	0.9	0.282
**Group 1 - DT (msec)**			
0–14	215.9 (183.6;253.9)	0 (ref).	-
15–180	212.1 (179.8;250.1)	-3.9	0.123
181–365	210.8 (179.6;247.4)	-5.1	0.361
366–545	203.6 (172.6;240.3)	-12.4	0.768
> 545	206.4 (174.9;244)	-9.5	0.675
**Group 1 - E/e’**			
0–14	6.6 (5.6;7.7)	0 (ref).	-
15–180	6.5 (5.6;7.6)	-0.1	0.329
181–365	6.8 (5.8;7.9)	0.2	0.838
366–545	7 (6;8.2)	0.4	0.402
> 545	6.8 (5.8;8)	0.2	0.869
**Group 2 - EF (%)**			
0–14	65.5 (62.5;68.6)	0 (ref).	-
15–180	63.5 (60.5;66.6)	-2.0	0.093
**181–365**	**62.1 (59;65.3)**	**-3.4**	**0.007**
366–545	64.4 (60.9;67.9)	-1.2	0.415
> 545	64.6 (61.2;68.1)	-0.9	0.51
**Group 2 - DT (msec)**			
0–14	227.9 (194;267.5)	0 (ref).	-
**15–180**	**197.4 (168;231.8)**	**-30.6**	**0.028**
181–365	206.2 (174.2;244.2)	-21.7	0.159
366–545	208.9 (171.9;253.9)	-19.0	0.294
> 545	209.6 (173.6;253.2)	-18.4	0.285
**Group 2 - E/e’**			
0–14	6.7 (5.8;7.8)	0 (ref).	-
15–180	7.1 (6.1;8.3)	0.4	0.3
181–365	7 (6;8.3)	0.3	0.5
366–545	7.6 (6.4;9.1)	0.9	0.1
> 545	7 (5.8;8.5)	0.3	0.6

Estimated marginal means based on linear mixed models. *p* < 0.05 was considered statistically significant. Group 1 included patients who received taxane-based chemotherapy treatment without anthracycline. Group 2 included patients who received anthracycline followed by taxane-based treatment. EMM, estimated marginal mean; 95% CI, 95% confidence interval; EF, ejection fraction; DT, deceleration time; E/ e’, ratio of early diastolic flow peak velocity of the mitral valve (E) and early diastolic peak velocity of mitral valve annulus (e’). First column shows the number of days since first chemotherapy. Column Diff shows the difference between EMMs of the given vs. the reference period (0–14 days). *Model 2* was adjusted for age, body mass index, hypertension, diabetes mellitus, hyperlipidaemia, current smoking, major adverse cardiac events, and previous chemotherapy as fixed effects

**Fig. 2:**
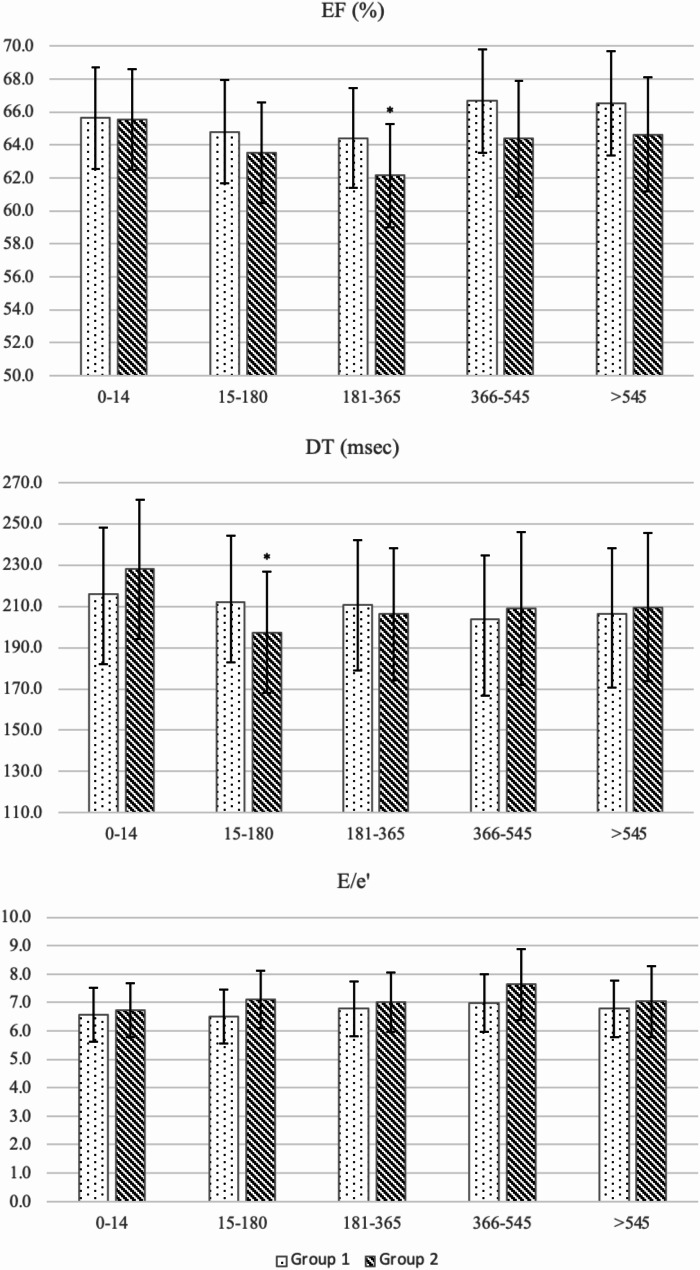
Estimated marginal means (with 95% confidence intervals) of EF, DT, E/e’ by type of treatment based on *Model 2* over the follow-up periods. Results based on linear mixed models. Error bars represent 95% confidence intervals. Model 2 was adjusted for age, body mass index, hypertension, diabetes mellitus, hyperlipidaemia, current smoking, major adverse cardiac events, previous chemotherapy as fixed effects. *p* < 0.05 was considered as statistically significant. Asterisk (*) sings periods with estimated marginal means significantly different from those in the reference period (1–14 days). Group 1 included patients who received taxane-based chemotherapy treatment without anthracycline. Group 2 included patients who received anthracycline followed by taxane-based treatment. EF, ejection fraction; DT, deceleration time; E/ e’, ratio of early diastolic flow peak velocity of the mitral valve (E) and early diastolic peak velocity of mitral valve annulus (e’)

In the anthracycline plus taxane group, the baseline estimated mean EF of 65.5% (95% CI: 62.5–68.6%) significantly decreased to 62.1% (95% CI: 59.0-65.3%, *p* = 0.007) in period 2, followed by an improvement in the later periods reaching 64.6% (95% CI: 62.1–68.1%, *p* = 0.510) in period 4 according to *Model 2*. Further adjustment for cardiovascular risk factors had no major effect on the observed U-shaped trajectories. (Table 2; Fig. 2) *Model 1* and *Model 3* gave similar results to those of *Model 2*. (Supplementary Table 1)

Other determinants of a lower EF were older age (mean difference [MD]: -0.1, 95% CI: -0.2;0.0 /year, *p* = 0.006) and the presence of hypertension (MD: -2.8, 95% CI: -4.8;-0.8, *p* = 0.006), which were associated with lower EF according to both *Model 1* and *Model 3*. (Table 3, Supplementary Table 2)

**Table 3:** Independent determinants of EF, DT, E/e’ in the anthracycline plus taxane group based on *Model 2*

	Effect size (95% CI)	*p*
**EF (%)**		
Age (year)	-0.1 (-0.2;0)	0.006
Hypertension	-2.8 (-4.8;-0.8)	0.006
**DT (msec)**		
-	-	-
**E/e’**		
Age (year)	1 (1;1)	< 0.001
BMI (kg/m^2^)	1 (1;1)	0.044
Hypertension	0.8 (0.7;0.9)	0.002
Diabetes	0.8 (0.7;1)	0.021

Results based on linear mixed models. *p* < 0.05 was considered statistically significant. 95% CI, 95% confidence interval; EF, ejection fraction; DT, deceleration time; E/ e’, ratio of early diastolic flow peak velocity of the mitral valve (E) and early diastolic peak velocity of mitral valve annulus (e’). *Model 2* was adjusted for age, body mass index, hypertension, diabetes mellitus, hyperlipidaemia, current smoking, major adverse cardiac events, previous chemotherapy as fixed effects

DT in the anthracycline plus taxane group also showed a U-shaped trajectory over time; however, it reached its lowest point in period 1. The estimated mean DT decreased from 227.9 (95% CI: 194.0-267.5) at baseline to 197.4 (95% CI: 168.0-231.8) msec in period 1 (*p* = 0.028) according to *Model 2*. (Table 2; Fig. 2) *Model 1* and *Model 2* gave similar results. Given that none of investigated cardiovascular risk factors in *Model 2* were independently associated with DT, no *Model 3* is presented. (Supplementary Table 1)

Although E/e’ did not change significantly during follow-up (Table 2, Fig. 2, Supplementary Table 1), we found that older age (estimated marginal means: 1.0, 95% CI: 1.0;1.0, p < 0.001), higher BMI (estimated marginal means: 1.0, 95% CI: 1.0;1.0, p = 0.044), the presence of hypertension (estimated marginal means: 0.8, 95% CI: 0.7;0.9, p = 0.002) and diabetes mellitus (estimated marginal means: 0.8, 95% CI: 0.7;1.0, p = 0.021) were associated with higher E/e’ values in *Model 2* (Table 3). Although diabetes was not a significant determinant according to *Model 3* (*p* = 0.095), the observed effect size was similar. (Supplementary Table 2)

Our sensitivity analysis (after the exclusion of patients not receiving concurrent anti-HER2 therapy) shows similar findings to our main analysis. In the single taxane group, no changes compared to baseline were found in any of the TTE parameters over follow-up. In the anthracycline plus taxane group, EF decreased from 65.7% (95% CI: 63.7–67.8%) to 62.6% (95% CI: 60.6–64.4%, p = 0.020) in period 1 and decreased further to 61.9% (95% CI: 59.9–63.9%, p = 0.004) in period 2. DT decreased from 233.0 (95% CI: 209.35–259.0) at baseline to 194.0 (95% CI: 174.5-215.7, p = 0.012) in period 1. E/e’ values showed no changes over time. (Supplementary Table 3)

## Discussion

In this retrospective study, we analysed TTE parameters in women with breast cancer treated with single taxane (80 mg/m^2^ paclitaxel) or anthracycline (mean dose 240 mg/m2 doxorubicin equivalent) plus taxane based regimens. We used EF defined by TTE as a marker of systolic function, while DT and the ratio of E and e’ (E/e’) were used to estimate diastolic function of the heart. According to the ESC guideline ECG testing is recommended only before treatment (recommendation level I), but not afterwards [14], so ECG data was not analysed.

In choosing which risk factors to use as fixed effects in linear mixed model, we followed the ESC guideline [14]. The guideline specifically addresses risk factors that should be considered before starting anthracycline-based treatment, but the same recommendations are frequently applied to patients starting taxane-based treatment. These risk factors include heart failure (cardiomyopathy), cancer therapy-related cardiac dysfunction (very-high risk), severe valvular heart disease, prior myocardial infarction, coronary revascularisation, stable angina, left ventricular ejection fraction < 50%, age > 80 years, radiotherapy to the left chest or mediastinum, and previous anthracycline therapy (high risk), as well as left ventricular ejection fraction 50–54%, age 65–79 years, increased cardiac troponin or natriuretic peptide levels, presence of hypertension, chronic kidney disease, diabetes mellitus, past/current smoking, and obesity (medium risk). None of the very-high risk states were present in this study, as they would preclude receipt of the studied chemotherapy regimens.

By using linear mixed models, we found a temporary and clinically insignificant decrease of EF in patients on anthracycline plus taxane chemotherapy during 181–365 days after baseline. After this period, EF improved and was similar to the baseline value. (Table 2; Fig. 2) Other important determinants of EF were older age and the presence of hypertension among the known cardiovascular risk factors according to *Model 2* (thus not related to previous MACE, previous chemotherapy, BMI, diabetes mellitus, hypercholesterolemia, smoking). (Table 3)

For markers related to diastolic function, DT showed a statistically significant, but clinically non-significant decrease in period 1 (15–180 days after treatment) compared to baseline. In the following periods, values improved and became similar to baseline. None of the investigated cardiovascular risk factors were independently related to DT. For E/e’, we found no significant alterations over time. (Table 2, Fig. 2) According to *Model 2*, independent determinants of E/e’ were older age, higher BMI, presence of hypertension, and diabetes. (Table 3)

No changes were detected in the measured cardiac functions in patients treated with single taxane chemotherapy. The alterations observed in measured TTE parameters were neither statistically nor clinically significant. (Table 2) This group included 12 patients with previous (beyond 365 days) cardiotoxic chemotherapy, which is considered as a high-risk risk factor for the development of cardiovascular events according to the ESC guideline [14]. Of the 12 patients, 10 received anthracycline chemotherapy and 6 received anti-HER2 therapy. As taxane chemotherapy was not associated with any changes in any of the TTE parameters tested, no further tests were run in this subgroup.

As most patients were on concurrent anti-HER2 treatment (40/45 patients in the single taxane group and 18/23 patients in the anthracycline plus taxane group) we analysed our data by excluding those 5 patients not on anti-HER2 therapy in both groups. This sensitivity analysis reinforces our findings in the main analysis showing (1) no changes in any TTE parameters in the single taxane group over time, and (2) statistically (but not clinically) significant temporary decreases in ejection fraction with nadirs in period 2 and in deceleration time in period 1 in the anthracycline plus taxane group compared to baseline. (Supplementary Table 3)

As the incidence, prevalence and survival rate of breast cancer improves [2], it becomes more important to follow-up patients for potential side effects of anti-cancer agents. Among them anthracyclines and taxanes have special interest, as they have high value in selected clinical situations, and they are frequently used medications.

With single taxane chemotherapy, no severe cardiac side effects are expected [20] and taxane may be safer in patients with pre-existing left ventricular dysfunction in whom anthracyclines should be avoided [21]. During single taxane therapy, regular monitoring of cardiac function is not recommended by the ESC Guidelines [14]. However, combinations of taxane and anthracyclines requires special attention as taxanes reduce the elimination of doxorubicin, leading to higher plasma levels [22]. According to a meta-analysis, the addition of taxane chemotherapy to anthracycline based regimens does not increase cardiotoxicity [23]. In this setting, paclitaxel seems to be more cardiotoxic than docetaxel [24]. Furthermore, docetaxel in combination with or even after anthracyclines may also increase the incidence of heart failure [25]. Our findings that taxanes are not associated with clinically significant alterations of TTE parameters of systolic and diastolic function are in line with the above literature and extend these to even those patients that have a history of cardiotoxic chemotherapy.

On the contrary, anthracyclines have long been recognized as cardiotoxic agents [26], with approximately 1% of unexplained cardiomyopathy cases related to anthracyclines [27]. Acute anthracycline toxicity is uncommon, often asymptomatic, and manifested as arrhythmias, pericarditis, or myocarditis [28]. In line with this, our study sample did not show evidence of acute toxicity. Furthermore, our study does not support the presence of clinically significant systolic and diastolic dysfunction even on longer term follow-up. However, subacute or chronic forms of anthracycline-related cardiotoxicity are more frequent and needs attention. In one study, early-onset chronic progressive anthracycline toxicity appeared in 20–30% of patients with new left ventricular dysfunction on imaging and 1.6–2.1% with symptomatic heart failure during treatment. It has to be noted that anthracycline-induced cardiotoxicity associated mortality did not differ in this study from that of a matched idiopathic dilatative cardiomyopathy [29]. Also, it has been shown that delayed onset (e.g. years after treatment) cardiac dysfunction may be relatively refractory to heart failure treatment and associated with a poor prognosis [30].

A systematic review and meta-analysis of randomized controlled trials showed a pronounced effect of anthracycline-based therapies not only on cardiac function but also on cardiac death [11]. Another study found that the overall incidence of anthracycline cardiotoxicity was 9% and the median time between end of chemotherapy and cardiotoxicity development was 3.5 months. In line with our observations this study also suggested that anthracyclines related cardiac side effects develop within the first year of treatment [12]. In a randomized study, subclinical impairment of cardiac function persisted over 2 years after the end of anthracycline-based treatment protocols [13]. In untreated cases, progressive decline of left ventricular function and a subsequent heart failure was observed [12]. Multiple studies have shown that the risk of heart failure rises with increasing cumulative doses [31, 32].

Age is also an important factor of heart failure in breast cancer patients. Various studies found that patients over 65 years or 76 years of age have an increased incidence of congestive heart failure compared to younger ones [31, 33]. In contrast, in our study, EF and DT recovered in patients on anthracycline-based regimen after a temporary, and clinically non-significant drop, which could be explained by a few different factors. First and foremost, the average age of our patients was younger than in the aforementioned studies and age is known to be a remarkable risk factor for cardiovascular diseases; the severity of cardiotoxicity was mainly dependent on the age of the patients (31; 33). The repair and regeneration capacity of the heart is limited, meaning older individuals have a greater tendency to develop cardiac disorders [34].

It has also been pointed out that for patients with preexisting cardiovascular comorbidities undergoing different chemotherapy regimens [35] or specifically anthracycline based treatments [36–38], it is of utmost importance to provide optimal cardiovascular treatment to avoid the development of early myocardial dysfunction induced by the chemotherapy. Our patients had a relatively low number of CV risk factors, therefore their overall cardiovascular burden was low. None of them had congestive heart failure at or before baseline according to their medical records. It should also be noted that chemotherapy protocols could differ in different studies meaning different associated cardiovascular risks. For example, trastuzumab treatment augments the cardiotoxic effects of anthracyclines [39]. Therefore, direct comparison of these studies with our results is limited. However, our data is reassuring as it suggests that in younger patients with low CV risk burden breast cancer treatment even with an anthracycline is relatively safe and its non-treatable cardiotoxic side effects are not common.

On the other hand, there are emerging methods that may be more sensitive to detect cardiac damage or may be earlier markers of future changes of cardiac function in patients receiving cardiotoxic chemotherapies. Measuring global longitudinal strain (GLS) with speckle tracking echocardiography could provide a valuable tool for the early detection of cardiac dysfunction even among patients with normal left ventricular EF. Despite its known limitations, completion of a GLS measurement is recommended for patients with moderate or high cardiovascular risk at baseline. However, if serial GLS measurements are planned, it should be performed using the same machine/software by the same investigator [14]. For the time being, GLS measurement is not yet available in our department in routine practice, but it may be a valuable tool for the adjustment of cardiovascular therapy in the future. Elevation of cardiac biomarkers (e.g. troponin T, natriuretic peptides) is useful to identify both clinical and subclinical forms of anthracycline-induced cardiotoxicity. These biomarkers have a reasonably high negative predictive value, but their use in clinical practice without typical symptoms (mainly chest pain, dyspnoea, or syncope) is not recommended presently in our clinic. Furthermore, cardiac MRI is also a valuable tool for detecting subclinical changes early after the beginning of anthracycline based therapy [40], but its routine clinical usage is limited by its high cost and time, as well as sparse availability.

The mechanisms of anthracycline associated cardiotoxicity are complex and a matter of debate. There are various signalling mechanisms involved in doxorubicin cardiotoxicity, including oxidative stress and cardiac mitochondrial damage, which may present within a few hours following the administration of doxorubicin [41]. It is also well known that oxidative stress plays a critical role in the aging process [42]. Anthracycline induces ultrastructural damage to cardiomyocytes, which eventually appears due to accumulated damage. Vacuolization of the cytoplasm and loss of myofibrils are characteristic features of the myocardium undergoing fibrosis and inflammation [42]. However, it has to be noted that the aging myocardium also acquires deleterious structural changes, including progressive cardiomyocyte hypertrophy, interstitial fibrosis, and inflammation, ultimately contributing to diastolic and systolic dysfunction [43]. A study directly comparing the alterations found in cardiomyocytes due to aging and anthracycline-based chemotherapy has yet to be completed.

Our study has considerable weaknesses that must be addressed. A large proportion of cases were excluded because their baseline cardiological evaluations were performed in other clinics. While this resulted in the exclusion of 152 patients, we think that this mostly affects patients who live further away from the oncology centre and thus it is unlikely to bias our findings. Another large proportion of exclusions is related to missing follow-up visits (*n* = 146) that could indeed cause selection bias although these patients had similar baseline characteristics to included patients. On the other hand, before starting the treatment, the mandatory cardiological examination was often performed elsewhere, so some patients reported to our outpatient clinic for the first time at a control visit. The time-points and frequencies of the recorded patients’ visits showed a great degree of variability. The follow-up of the patients, the initiation of therapy in case of tumour relapse, and the cardiological control before the change of therapy were always completed, but not necessarily at our clinic and therefore several patients’ data was excluded. Although included and excluded patients were similar in terms of investigated characteristics, given the high exclusion rate, we cannot exclude the role of unmeasured confounders and a potential selection bias cannot be excluded. Other regular follow-up investigations were used dependent on patients’ requests and compliance. Based on all of this, individual visits could follow each other from every few weeks to several years and for this reason, we used the above wide periods of follow-up in our modelling. Given that most patients received anti-HER2 in addition to their chemotherapy regimen, we were unable to control for its effect in our analysis. However, the observation that anti-HER2 therapy had no effect on the observed echocardiographic parameters in the taxane therapy group strongly suggests that our findings in the anthracycline group are reflecting anthracycline related changes.

Our study has strengths that has to be acknowledged. While our study has excellent internal validity, given that only a limited number of expert echocardiographers performed all investigations using standard protocols, the measures we collected are widely used in routine clinical practice and thus the external validity of the findings is excellent. Given that the ESC guideline considers taxane treatment safe, it is rarely investigated in the literature. However, the local protocol at our institution requires similarly close follow-up to anthracycline therapies, and thus our study benefits from the presence of a negative control group. Also, the use of the gold standard statistical method for repeat data improves the sensitivity to detect mild alterations in a relatively small population.

In summary, we found that anthracyclines caused temporary and clinically non-significant deteriorations in the EF and the DT in our relatively young patient population treated for breast cancer, while E/e’ did not change significantly. Also, in line with recent guidelines, taxane treatment was found to be safe from a cardiological standpoint even in patients that received previous anthracycline chemotherapy (considered high risk factor for most chemotherapy regimens) or concurrent anti-HER2 therapy. Our results overall confirm the recommendations of the ESC cardio-oncology guideline that considers taxane treatment low risk and the recommended intensity of TTE follow-up is based on overall baseline risk assessment.

The pattern of changes suggests a deterioration in diastolic function first, with changes in EF thereafter. This observation points to the need for follow up by monitoring not just EF alone, as changes in diastolic function may be an early warning sign. Low risk factors according to the ESC guideline [14] (such as diabetes mellitus, hypercholesterolemia and smoking) were not significant determinants of diastolic or systolic function in our study. For both EF and E/e’, age (according to the ESC guideline high or medium risk, depending on its value) and hypertension (low risk in the ESC guideline) were independent determinants [14]. Further investigations and follow-up studies are required to better determine characteristics of patients that will suffer clinically significant deterioration as a consequence of anthracycline therapies.

## Electronic supplementary material

Below is the link to the electronic supplementary material.

Supplementary Material 1

## Data Availability

No datasets were generated or analysed during the current study.
