# Supplementary material for: Effects of taxane-anthracycline and taxane only treatment on cardiac function in breast cancer—a retrospective cohort study
Source: Cardiooncology. 2025 Apr 12;11:37. doi: 10.1186/s40959-025-00335-4 (PMC11992891; doi:10.1186/s40959-025-00335-4)
Supplement: Supplementary file 1 — Supplementary Material 1 [file 40959_2025_335_MOESM1_ESM.docx]

**Supplementary Table 1.** Estimated marginal means of EF, DT, E/e' by treatment type based on *Model 1* and *Model 3* over the follow-up periods

| **Days** | **Model 1** | | | **Model 3** | | |
| --- | --- | --- | --- | --- | --- | --- |
|  | **EMM (LCI;UCI)** | **Diff** | **p** | **EMM (LCI;UCI)** | **Diff** | **p** |
| **Group 1 - EF (%)** | | | | | | |
| 0-14 | 64.3 (63;65.7) | 0 (ref.) | - | 64.8 (63.4;66.1) | 0 (ref.) | - |
| 15-180 | 63.4 (61.9;64.8) | -0.9 | 0.495 | 64 (62.5;65.4) | -0.8 | 0.421 |
| 181-365 | 63 (61.8;64.3) | -1.3 | 0.149 | 63.6 (62.3;64.9) | -1.2 | 0.131 |
| 366-545 | 65.1 (63.6;66.6) | 0.8 | 0.152 | 65.9 (64.3;67.4) | 1.1 | 0.183 |
| >545 | 64.7 (63.1;66.2) | 0.3 | 0.577 | 65.7 (64.1;67.3) | 1.0 | 0.198 |
| **Group 1 - DT (msec)** | | | | | | |
| 0-14 | 212.5 (198.3;227.7) | 0 (ref.) | - | - | - | - |
| 15-180 | 208.9 (193.6;225.4) | -3.6 | 0.104 | - | - | - |
| 181-365 | 207.7 (194.6;221.6) | -4.8 | 0.293 | - | - | - |
| 366-545 | 201.7 (186.6;217.9) | -10.8 | 0.739 | - | - | - |
| >545 | 204.6 (189.6;221) | -7.9 | 0.329 | - | - | - |
| **Group 1 - E/e'** | | | | | | |
| 0-14 | 6.5 (1.8;2) | 0 (ref.) | - | 6.8 (6.3;7.4) | 0 (ref.) | - |
| 15-180 | 6.4 (1.8;2) | 0.0 | 0.279 | 6.8 (6.2;7.4) | -0.1 | 0.316 |
| 181-365 | 6.7 (1.8;2) | 0.2 | 0.947 | 7 (6.5;7.7) | 0.2 | 0.904 |
| 366-545 | 6.9 (1.8;2) | 0.4 | 0.488 | 7.2 (6.6;7.9) | 0.4 | 0.357 |
| >545 | 6.8 (1.8;2) | 0.4 | 0.907 | 7 (6.4;7.7) | 0.2 | 0.853 |
| **Group 2 - EF (%)** | | | | | | |
| 0-14 | 65 (63.2;66.9) | 0 (ref.) | - | 65.4 (63.5;67.2) | 0 (ref.) | - |
| 15-180 | 63.1 (61.2;65) | -2.0 | 0.101 | 63.3 (61.5;65.2) | -2.0 | 0.091 |
| **181-365** | **61.6 (59.6;63.6)** | -3.5 | **0.006** | **61.9 (59.9;63.9)** | **-3.5** | **0.006** |
| 366-545 | 63.4 (61;65.8) | -1.7 | 0.242 | 64.2 (61.8;66.6) | -1.2 | 0.412 |
| >545 | 64.4 (62.2;66.7) | -0.6 | 0.661 | 64.2 (62;66.4) | -1.2 | 0.382 |
| **Group 2 - DT (msec)** | | | | | | |
| 0-14 | 229.1 (207.7;252.9) | 0 (ref.) | - | - | - | - |
| **15-180** | **197.2 (178.8;217.7)** | **-31.9** | **0.022** | **-** | - | **-** |
| 181-365 | 205.2 (183.6;229.3) | -23.9 | 0.118 | - | - | - |
| 366-545 | 210.6 (183.5;242) | -18.5 | 0.306 | - | - | - |
| >545 | 202.4 (179.3;228.4) | -26.7 | 0.095 | - | - | - |
| **Group 2 - E/e'** | | | | | | |
| 0-14 | 6.4 (5.5;7.3) | 0 (ref.) | - | 6.9 (6.2;7.6) | 0 (ref.) | - |
| 15-180 | 6.8 (5.9;7.8) | 0.4 | 0.225 | 7.3 (6.6;8.1) | 0.4 | 0.284 |
| 181-365 | 6.6 (5.7;7.6) | 0.2 | 0.594 | 7.1 (6.4;8) | 0.3 | 0.518 |
| 366-545 | 7.2 (6.1;8.4) | 0.8 | 0.08 | 7.9 (6.9;9) | 1.0 | 0.051 |
| >545 | 6.8 (5.7;8.1) | 0.4 | 0.411 | 7.2 (6.2;8.4) | 0.3 | 0.548 |

Estimated marginal means based on linear mixed models. p < 0.05 was considered statistically significant. Group 1 included patients who received taxane-based chemotherapy treatment without anthracycline. Group 2 included patients who received anthracycline followed by taxane-based treatment. EMM, estimated marginal mean; EF, ejection fraction; DT, deceleration time; E/ e', ratio of early diastolic flow peak velocity of the mitral valve (E) and early diastolic peak velocity of mitral valve annulus (e'). First column shows the number of days since first chemotherapy. Column Diff shows the difference between EMMs of the given vs the reference period (0-14 days). *Model 1* shows unadjusted data. *Model 3* was adjusted for age and hypertension as fixed effects for EF and for age, body mass index, hypertension, and diabetes mellitus for E/e’.

**Supplementary Table 2.** Independent determinants of EF, DT, E/e' in the anthracycline plus taxane group based on *Model 3*

|  | **Effect size  (95% CI)** | **p** |
| --- | --- | --- |
| **EF (%)** | | |
| Age (year) | -0.1 (-0.2;0) | 0.006 |
| Hypertension | -3.1 (-4.8;-1.3) | <0.001 |
| **DT (msec)** | | |
| - | - | - |
| **E/e'** | | |
| Age (year) | 1 (1;1) | <0.001 |
| BMI (kg/m^2^) | 1 (1;1) | 0.01 |
| Hypertension | 0.9 (0.8;0.9) | 0.004 |
| Diabetes | 0.9 (0.8;1) | 0.095 |

Results based on linear mixed models. p < 0.05 was considered statistically significant. 95% CI, 95% confidence interval; EF, ejection fraction; DT, deceleration time; E/ e', ratio of early diastolic flow peak velocity of the mitral valve (E) and early diastolic peak velocity of mitral valve annulus (e'). *Model 2* was adjusted for age, body mass index, hypertension, diabetes mellitus, hyperlipidaemia, current smoking, major adverse cardiac events, previous chemotherapy as fixed effects. *Model 3* was adjusted for age and hypertension as fixed effects for EF and for age, body mass index, hypertension, and diabetes mellitus for E/e’.

**Supplementary Table 3.** Estimated marginal means of EF, DT, E/e' by treatment type restricted to patients who received concomitant anti-HER2 therapy based on *Model 1* over the follow-up periods

| **Days** | **EMM (LCI;UCI)** | **Diff** | **p** |
| --- | --- | --- | --- |
| **Group 1 - EF (%)** | | | |
| 0-14 | 64.9 (63.5;66.3) | 0 (ref) | - |
| 15-180 | 63.8 (62.3;65.2) | -1.1 | 0.22 |
| 181-365 | 63.4 (62.1;64.6) | -1.5 | 0.139 |
| 366-545 | 65.3 (63.8;66.8) | 0.4 | 0.136 |
| >545 | 64.8 (63.4;66.3) | -0.1 | 0.47 |
| **Group 1 - DT (msec)** | | | |
| 0-14 | 218.1 (203.0;234.2) | 0 (ref) | - |
| 15-180 | 209.3 (193.6;226.1) | -8.8 | 0.108 |
| 181-365 | 212.3 (198,9;226.6) | -5.8 | 0.231 |
| 366-545 | 205.2 (190.0;221.8) | -12.9 | 0.693 |
| >545 | 205.6 (190.9;221.6) | -12.5 | 0.368 |
| **Group 1 - E/e'** | | | |
| 0-14 | 6.4 (5.8;7.0) | 0 (ref) | - |
| 15-180 | 6.4 (5.8;7.2) | 0.0 | 0.455 |
| 181-365 | 6.6 (6.0;7.3) | 0.2 | 0.941 |
| 366-545 | 6.8 (6.1;7.5) | 0.2 | 0.766 |
| >545 | 6.8 (6.1;7.5) | 0.0 | 0.936 |
| **Group 2 - EF (%)** | | | |
| 0-14 | 65.7 (63.7;67.8) | 0 (ref) | - |
| **15-180** | **62.6 (60.6;64.6)** | **-3.1** | **0.02** |
| **181-365** | **61.9 (59.9;63.9)** | **-3.8** | **0.004** |
| 366-545 | 63.5 (61.1;66.0) | -2.2 | 0.143 |
| >545 | 64.5 (62.3;66.7) | -1.2 | 0.364 |
| **Group 2 - DT (msec)** | | | |
| 0-14 | 233.0 (209.3;259.0) | 0 (ref) | - |
| **15-180** | **194.0 (174.5;215.7)** | **-39.0** | **0.012** |
| 181-365 | 204.4 (183.5;227.9) | -28.6 | 0.075 |
| 366-545 | 210.8 (182.7;243.2) | -22.2 | 0.251 |
| >545 | 202.6 (180.4;227.7) | -30.4 | 0.066 |
| **Group 2 - E/e'** | | | |
| 0-14 | 6.1 (5.2;7.2) | 0 (ref) | - |
| 15-180 | 6.5 (5.6;7.7) | 0.4 | 0.263 |
| 181-365 | 6.3 (5.4;7.4) | 0.2 | 0.593 |
| 366-545 | 6.7 (5.6;8.0) | 0.6 | 0.227 |
| >545 | 6.6 (5.4;7.9) | 0.5 | 0.394 |

Estimated marginal means based on linear mixed models. p < 0.05 was considered statistically significant. Group 1 included patients who received taxane-based chemotherapy treatment without anthracycline. Group 2 included patients who received anthracycline followed by taxane-based treatment. EMM, estimated marginal mean; EF, ejection fraction; DT, deceleration time; E/ e', ratio of early diastolic flow peak velocity of the mitral valve (E) and early diastolic peak velocity of mitral valve annulus (e'). First column shows the number of days since first chemotherapy. Column Diff shows the difference between EMMs of the given vs the reference period (0-14 days). *Model 1* shows unadjusted data.
